# Effect of Mineral Composition on CO_2_ Mineralization and Mechanical Strengthening of Synthetic Tailings

**DOI:** 10.3390/gels12090802

**Published:** 2026-09-02

**Authors:** Wei Lu, Shaoping Ren, Yao Lu, Zhi Guo, Changxiang Wang

**Affiliations:** 1 State Key Laboratory for Safe Mining of Deep Coal Resources and Environment Protection, Anhui University of Science and Technology, Huainan 232000, China; 2 School of Safety Science and Engineering, Anhui University of Science and Technology, Huainan 232000, China

**Keywords:** synthetic tailings, all-component compounding, carbonate precipitation, CO_2_ mineralization, mechanical strengthening

## Abstract

To elucidate the influence of mineral composition on CO_2_ mineralization and mechanical reinforcement, three synthetic tailing systems representing felsic, high-calcium silicate, and magnesium-carbonate compositions were prepared using a full-component reconstitution approach. Their carbonation kinetics, phase evolution, pore structure, and mechanical response were systematically investigated. The alkaline buffering duration increased in the order of felsic < high-calcium silicate < magnesium-carbonate types, with plateau durations of approximately 300, 650, and 2000 s, respectively. TG-DTG, XRD, and FTIR analyses collectively indicated composition-dependent formation of carbonate-bearing products, with the magnesium-carbonate type showing the strongest carbonate-related signals. SEM observations further revealed precipitation on particle surfaces and within interparticle regions, suggesting precipitation-induced particle bonding. Nitrogen adsorption–desorption measurements showed pronounced pore-structure reorganization after mineralization; the average pore size converged to approximately 7.5–7.9 nm, whereas the specific surface area decreased in all systems. The failure loads of the felsic and high-calcium silicate types increased by factors of 2.97 and 3.81, respectively. The mineralized magnesium-carbonate type exhibited the highest apparent compressive strength (53.69 kPa). These results demonstrate clear composition-dependent relationships among carbonation, pore evolution, and mechanical response in the synthetic systems. Because the materials were prepared from analytical-grade reagents and no complete carbon balance was established, the results should be regarded as mechanistic model-system evidence rather than quantitative estimates of CO_2_ sequestration in natural tailings.

## 1. Introduction

With the rapid development of China’s mining and mineral processing industry, large quantities of tailings have been generated and accumulated, making tailings one of the major categories of industrial solid waste in China [1]. The cumulative stockpile of tailings in China has exceeded 10 billion tons, with annual generation reaching approximately 1.2 billion tons. However, the comprehensive utilization rate remains relatively low, with only about 13.3% of tailings being effectively utilized [2]. Long-term accumulation and improper management of tailings can lead to environmental risks and safety hazards [3,4,5] and adversely affect surrounding ecosystems [6,7,8,9]. Therefore, developing effective approaches for tailings utilization while improving their mechanical and physicochemical performance is important for both tailings management and resource recovery.

Tailings backfilling and related disposal technologies have been increasingly applied to reduce the risks associated with tailings accumulation [10,11]. Nevertheless, conventional backfilling does not fully address the potential for tailings resource utilization or provide an effective pathway for carbon sequestration. In this context, CO_2_ mineralization based on the chemical composition of tailings has emerged as a promising approach. This process is analogous to the natural weathering of silicate minerals and enables alkaline-earth metal-bearing components to react with dissolved CO_2_ to form stable carbonate products [12,13]. In particular, Ca- and Mg-bearing minerals can provide reactive cations for the formation of carbonate phases such as CaCO_3_ and MgCO_3_. Consequently, CO_2_ mineralization offers the potential to simultaneously utilize tailings and achieve long-term CO_2_ sequestration [14]. Several studies have explored this concept using different solid-waste systems. Justin A et al. [15] coupled organic and inorganic carbon cycles to accelerate brucite carbonation in ultramafic tailings and investigated the effects of particle size and organic matter content on carbonation and associated cementation. Héctor et al. [16] investigated the effects of calcium and urea dosages on microbial-induced carbonate precipitation (MICP) for tailings biocementation and demonstrated its potential for reducing wind erosion. Wang et al. [17] chemically activated ultrabasic tailings using inorganic hydrogen sulfate and subsequently applied a two-stage carbonation process to mine backfilling, achieving a reported CO_2_ uptake of 13.46%.

Beyond CO_2_ sequestration, mineralization can also modify the structural and mechanical properties of solid-waste-based materials through the formation of carbonate products and associated microstructural changes [18,19,20]. Ma et al. [21,22] developed fly ash-based filling materials with both mechanical strength and mineralization-based CO_2_ storage capacity under room-temperature and atmospheric-pressure conditions using an alkali activator. Liu et al. [23] utilized the chemical complementarity of Ca, Si, Al, and S in multicomponent solid wastes to promote alkali activation of Si-Al-containing materials and coupled this process with sulfate reactions, thereby improving the cementation strength and durability of the resulting filling materials. Fan et al. [24] developed high-performance CO_2_ foam concrete (CFC) by incorporating CO_2_ into foam-based materials and demonstrated its potential for CO_2_ capture and storage. Xu et al. [25] proposed a coupled strategy integrating continuous backfilling, water-preserved coal mining, and CO_2_ mineralization and storage. In this approach, fly ash was used as the primary material, while CO_2_ and silicate-based cementitious components served as activation constituents to prepare mineralized filling materials under room-temperature and atmospheric-pressure conditions. In addition, gypsum carbonation has been investigated as another potential route for CO_2_ fixation, with experimental studies reporting a CO_2_ fixation capacity of approximately 228 kg per ton of phosphogypsum [26,27].

Despite these advances, the relationship between mineral composition, carbonation behavior, and mechanical strengthening remains insufficiently understood for tailing-like materials. Natural tailings contain complex and variable mineral assemblages, making it difficult to distinguish the intrinsic contribution of individual mineral components from the effects of particle characteristics, impurities, and coupled reactions. In particular, the extent to which different mineral compositions regulate alkaline buffering, carbonation reactivity, carbonate-product formation, pore-structure evolution, and subsequent mechanical response has not been systematically clarified. A controlled synthetic system with predefined mineral compositions can therefore provide a useful means of isolating composition-dependent effects while minimizing the compositional complexity of natural tailings.

Accordingly, this study employed a full-component reconstitution approach to construct three synthetic tailing systems representing typical felsic, high-calcium silicate, and magnesium-carbonate compositions. The carbonation behavior, thermal characteristics, reaction-product evolution, microstructural changes, pore-structure evolution, and mechanical responses of the three systems were systematically investigated using online pH monitoring, TG-DTG, XRD, FTIR, SEM, nitrogen adsorption, and mechanical testing. The objective was to clarify how mineral composition regulates carbonation behavior and how mineralization-induced product formation and structural reorganization are associated with mechanical reinforcement. By establishing the relationship between mineral composition, carbonation response, microstructural evolution, and mechanical performance, this study provides a controlled framework for understanding composition-dependent CO_2_ mineralization of tailing-like materials and contributes to the development of synergistic strategies for tailings utilization and CO_2_ sequestration.

## 2. Result and Discussion

The objective of this section is to clarify how carbonate-bearing precipitates and possible gel-like silicate/aluminosilicate products contribute to microstructural evolution and mechanical reinforcement during CO_2_ mineralization of three synthetic tailings systems with contrasting chemical compositions. Because the systems were prepared from analytical-grade reagents, the results are interpreted as model-system evidence for composition-structure-performance relationships rather than as direct quantitative predictions for natural tailings.

### 2.1. CO_2_ Mineralization Process and Product Thermogravimetric Analysis

The pH evolution and corresponding pH-change-rate curves of the three synthetic tailings systems were interpreted with reference to the hydration and carbonation reactions shown in Equations (1)–(6). Reactions (1)–(3) represent possible hydration pathways of alkaline components [28,29], whereas Reactions (4)–(6) describe subsequent CO_2_ dissolution, carbonate speciation, and carbonate precipitation processes [30].

(1) $$H_{2}O\to H^{+}+{OH}^{-}$$

(2) $$CaO+H_{2}O\to {Ca(OH)}_{2}$$

(3) $$MgO+H_{2}O⇌{Mg(OH)}_{2}$$

(4) $${CO}_{2}+H_{2}O⇌H_{2}{CO}_{3}⇌H^{+}+{HCO}_{3}^{-}$$

(5) $$H{CO}_{3}^{-}⇌H^{+}+{CO}_{3}^{2-}$$

(6) $$H_{2}{CO}_{3}+{Ca(OH)}_{2}/{Mg(OH)}_{2}\to {CaCO}_{3}/{MgCO}_{3}$$

**Figure 1:**
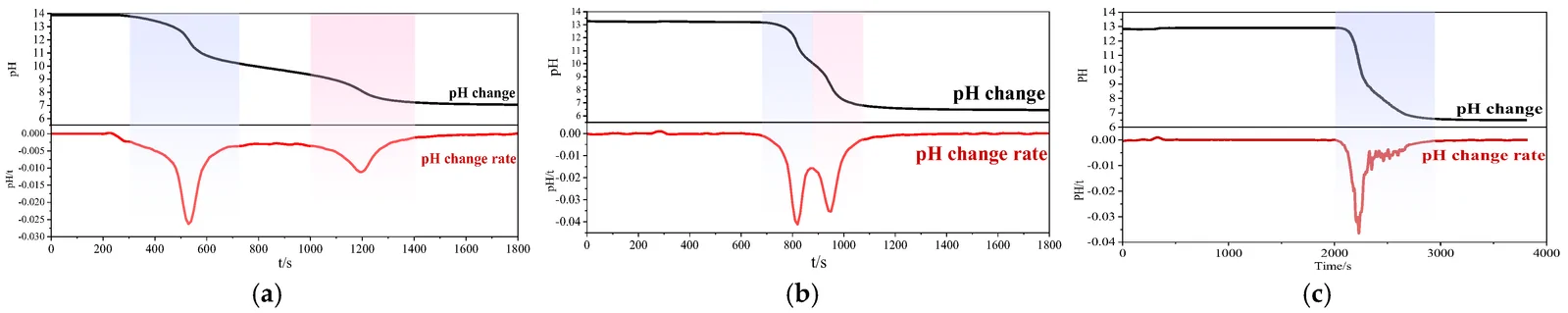
Mineralization time–pH characteristic curves of the three synthetic tailing systems: (**a**) felsic type, (**b**) high-calcium silicate type, and (**c**) magnesium-carbonate type.

As shown in Figure 1, the pH profiles differed markedly among the three systems. The felsic type showed the shortest alkaline plateau (approximately 300 s), followed by the high-calcium silicate type (650 s) and magnesium-carbonate type (2000 s). The initial pH decline was most pronounced for the high-calcium silicate type, followed by the magnesium-carbonate and felsic types. For the felsic system, the double negative peaks in the pH-change-rate curve may reflect two temporally distinguishable stages of alkalinity consumption, involving rapid CO_2_ dissolution and neutralization of readily available alkaline components followed by slower dissolution of Ca/Mg-bearing components and carbonate-bearing product precipitation [31]. Thus, the double-peak feature is interpreted as the result of sequentially coupled dissolution, neutralization, and precipitation processes rather than as evidence for two specific mineral phases. The prolonged alkaline period of the magnesium-carbonate type may be associated with the slower dissolution and carbonation of Mg-bearing components, whereas the rapid pH decline of the high-calcium silicate type indicates comparatively rapid alkalinity consumption. Overall, the pH-rate profiles reflect the combined effects of dissolution, hydration, neutralization, and precipitation processes.

**Figure 2:**
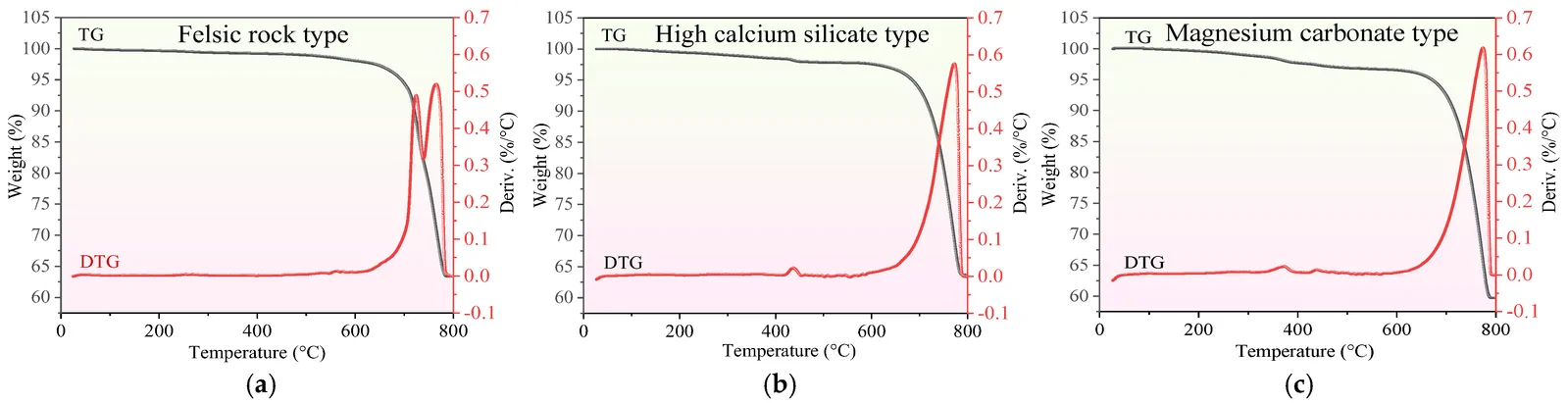
TG and DTG curves of mineralized products from the three synthetic tailing systems: (**a**) felsic type, (**b**) high-calcium silicate type, and (**c**) magnesium-carbonate type.

The TG-DTG profiles of the mineralized products are shown in Figure 2. All three systems exhibited limited mass loss below 600 °C, followed by more pronounced decomposition at higher temperatures. The apparent mass losses were approximately 36%, 37%, and 40% for the felsic, high-calcium silicate, and magnesium-carbonate types, respectively. The elevated-temperature mass-loss region is consistent with the decomposition of carbonate-bearing phases, although overlapping carbonate decomposition and dehydroxylation prevent direct phase-specific quantification. The stronger DTG response of the magnesium-carbonate type is consistent with its XRD and FTIR results, indicating a relatively greater abundance of carbonate-bearing products.

The TG values are therefore used only as comparative indicators of apparent mass loss associated with carbonate-bearing products and are not interpreted as direct CO_2_ uptake. Quantitative assessment of CO_2_ sequestration requires a complete carbon balance or an independently calibrated quantitative method. Accordingly, CO_2_ mineralization in this study refers to the observed formation of carbonate-bearing phases rather than quantified CO_2_ sequestration.

### 2.2. Phase Evolution During Hydration and CO_2_ Mineralization

XRD was used to identify crystalline phase changes before treatment, after hydration, and after CO_2_ mineralization. The hydrated products refer to samples reacted with water in the absence of CO_2_, whereas the mineralized products refer to samples treated with CO_2_ under aqueous conditions. Because XRD is mainly sensitive to crystalline phases, poorly crystalline or amorphous products may not be clearly detected; therefore, the results were interpreted together with FTIR and SEM observations.

**Figure 3:**
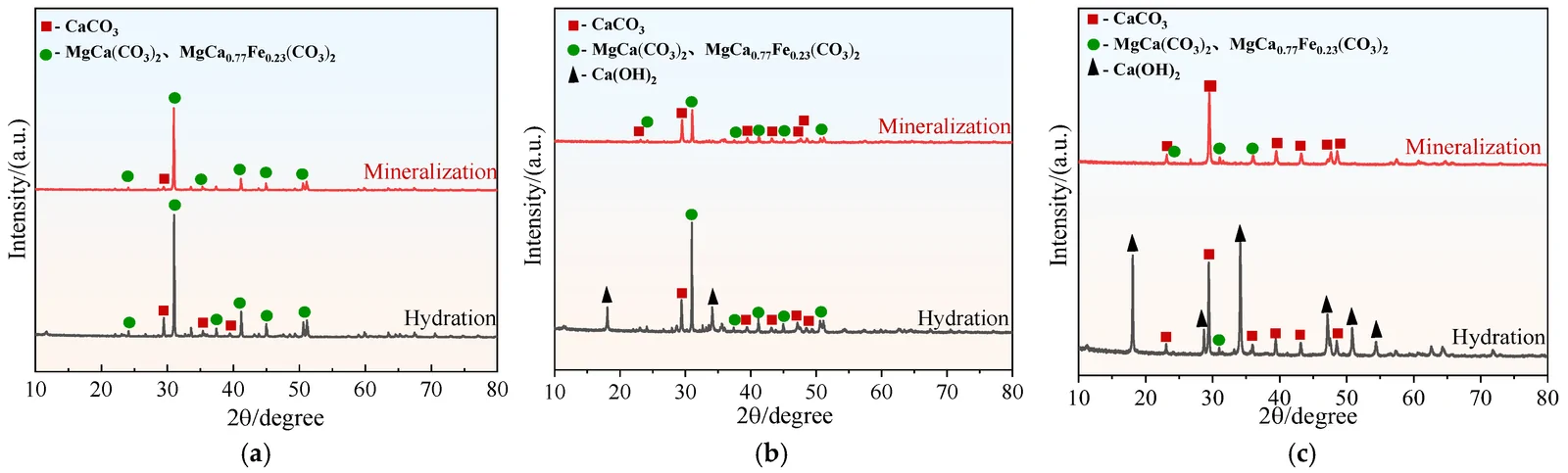
XRD patterns of hydration and mineralization products from the three synthetic tailing systems: (**a**) felsic type, (**b**) high-calcium silicate type, and (**c**) magnesium-carbonate type. Note: The PDF cards used for phase identification in this subsection are: calcite, PDF#00-005-0586, portlandite, Ca(OH)_2_, PDF#00-044-1481, and dolomite, PDF#00-011-0078.

As shown in Figure 3a, for the felsic-type system, only weak changes were observed after hydration, indicating limited formation of new crystalline hydration products. After CO_2_ mineralization, carbonate-related diffraction peaks increased only slightly, suggesting limited formation of crystalline carbonate-bearing products, consistent with its relatively low content of reactive Ca- and Mg-bearing components.

As shown in Figure 3b, the high-calcium silicate-type system exhibited more evident phase changes after hydration and CO_2_ mineralization. Its Ca-bearing components may provide Ca^2+^ for subsequent carbonate precipitation, consistent with the increased carbonate-related diffraction peaks after CO_2_ treatment. Compared with the felsic type, this system showed a more pronounced crystalline carbonate response.

As shown in Figure 3c, the magnesium-carbonate-type system exhibited the strongest carbonate-related crystalline response. The enhanced diffraction peaks after CO_2_ mineralization were consistent with its longer alkaline buffering period and greater apparent TG mass-loss response, suggesting relatively greater formation of carbonate-bearing crystalline products. However, this response does not represent a direct quantification of absolute CO_2_ uptake.

Overall, the crystalline phase evolution was strongly dependent on the designed chemical composition. Weak or absent diffraction peaks do not exclude poorly crystalline or amorphous products; FTIR was therefore used to provide complementary information on carbonate groups and silicate/aluminosilicate bonding environments.

### 2.3. FTIR Evidence for Carbonate and Possible Gel-like Products

FTIR spectroscopy was used to provide complementary information on functional groups and bonding environments. Unlike XRD, FTIR can detect spectral features associated with both crystalline and poorly crystalline materials, although it cannot independently determine exact phase identities. The main band assignments are summarized in Table 1 [32,33].

**Figure 4:**
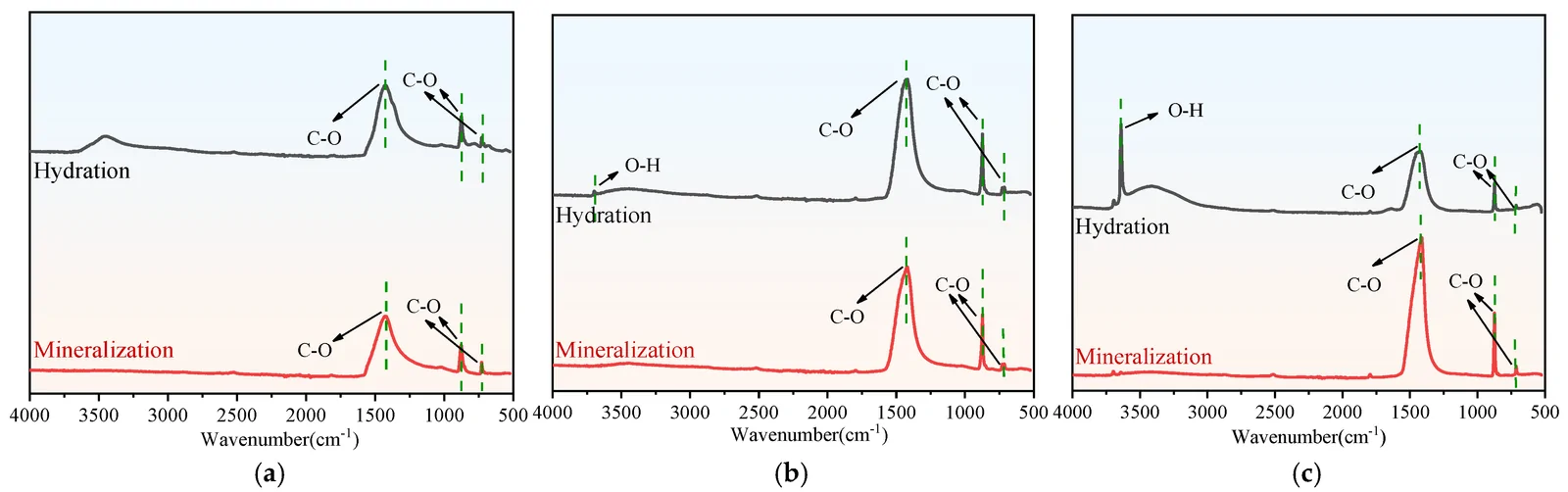
FTIR spectra of the three synthetic tailing systems before treatment, after hydration, and after CO_2_ mineralization: (**a**) felsic type, (**b**) high-calcium silicate type, and (**c**) magnesium—carbonate type.

As shown in Figure 4a, for the felsic-type system, carbonate-related FTIR bands after CO_2_ mineralization were relatively weak, consistent with the limited enhancement of carbonate-related XRD peaks. This indicates the weakest carbonate-formation response among the three systems.

As shown in Figure 4b, for the high-calcium silicate-type system, carbonate-related bands became more evident after CO_2_ mineralization, supporting the XRD evidence for carbonate-bearing products. Changes in the Si–O–T region further suggest modification of the silicate/aluminosilicate bonding environment and possible formation of poorly crystalline binding phases. However, these features cannot be assigned to specific C-S-H, C-A-S-H, or other gel phases based solely on FTIR.

As shown in Figure 4c, the magnesium-carbonate-type system showed the most pronounced carbonate-related FTIR response among the three systems, in agreement with its XRD and TG-DTG results. Variations in the Si–O–T region further suggest changes in the silicate/aluminosilicate bonding environment. However, the exact composition and structure of the possible poorly crystalline binding phases require further verification by complementary techniques such as SEM-EDS, NMR, XPS, or quantitative phase analysis.

Overall, the FTIR results support the composition-dependent carbonate-formation behavior identified by XRD and provide complementary evidence for interpreting the SEM-observed microstructural changes.

### 2.4. SEM-Observed Microstructural Evolution

SEM was used to compare the morphological changes between the hydrated and CO_2_-mineralized products. Because SEM was not coupled with EDS, the chemical composition of individual morphological features could not be directly determined. Therefore, SEM observations were interpreted primarily as morphological evidence and considered together with the XRD and FTIR results.

**Figure 5:**
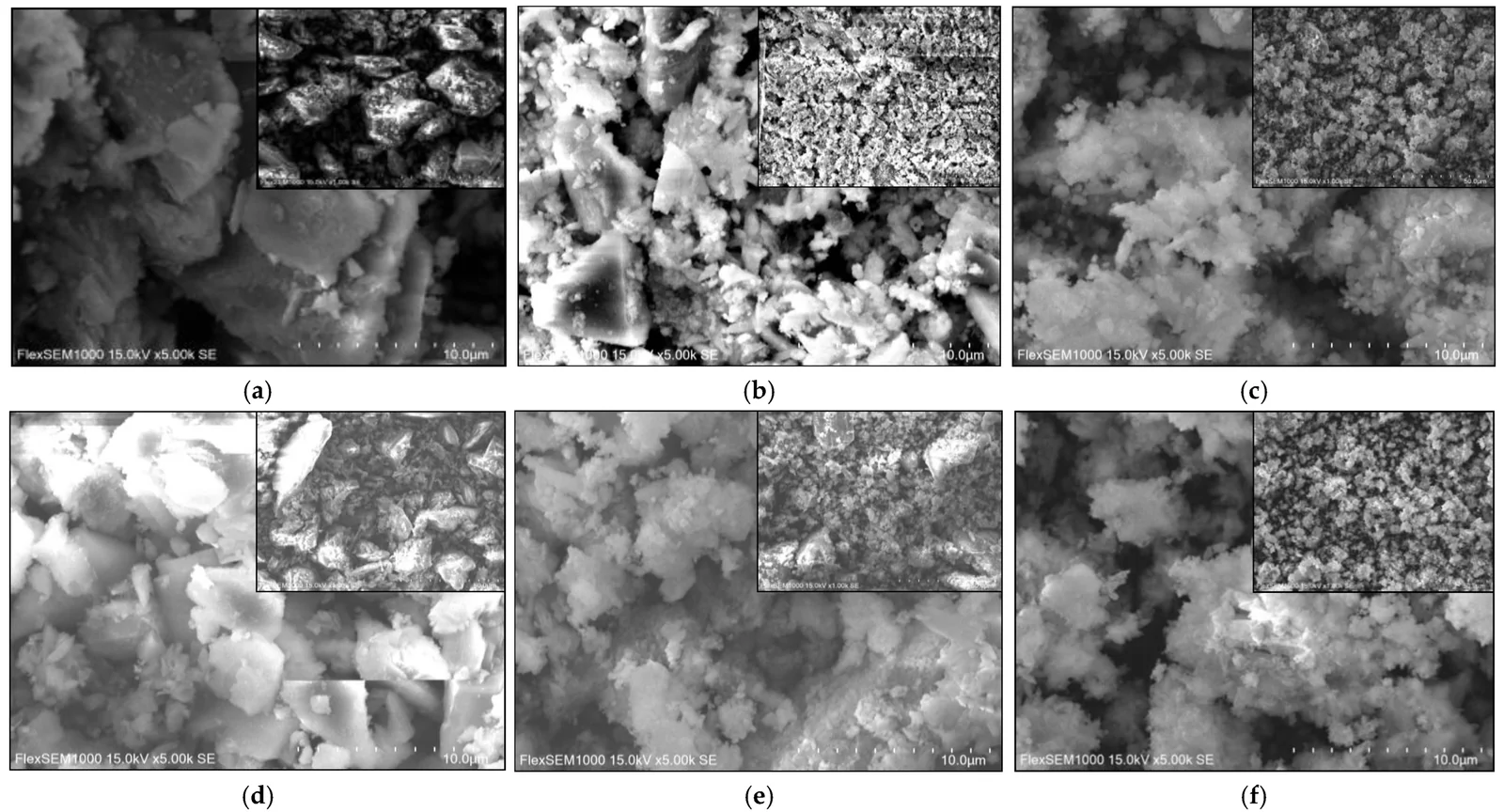
SEM images of the hydrated and mineralized products from the three synthetic tailing systems: hydrated products of the (**a**) felsic type, (**b**) high-calcium silicate type, and (**c**) magnesium-carbonate type; mineralized products of the (**d**) felsic type, (**e**) high-calcium silicate type, and (**f**) magnesium-carbonate type.

As shown in Figure 5a–c, the hydrated products generally exhibited relatively loose particle packing, with limited interparticle bonding [34]. After CO_2_ mineralization, varying degrees of surface coverage, local pore filling, and particle bridging were observed (Figure 5d–f). Similar precipitate-like morphologies and surface coatings have been reported in mineral carbonation and alkali-activated/cementitious systems [35,36]. In Mg-rich carbonation systems, carbonate precipitation on mineral surfaces and within interparticle regions has also been associated with microstructural modification and cementation effects.

For the felsic-type system, the hydrated product showed relatively loose particle arrangement and limited surface reaction features (Figure 5a). After CO_2_ mineralization, only minor surface coverage and local filling were observed (Figure 5d), consistent with its relatively weak carbonate-related XRD and FTIR responses. In contrast, the high-calcium silicate-type system exhibited more evident surface coverage and interparticle connections after mineralization (Figure 5e), consistent with its stronger carbonate-related XRD and FTIR responses. These observations suggest that carbonate-bearing products, together with possible poorly crystalline silicate/aluminosilicate products, were associated with increased interparticle contact and local pore filling. Similar reaction-product morphologies have been reported in Ca-rich alkali-activated and cementitious systems.

The magnesium-carbonate-type system showed the most pronounced morphological changes. Compared with the hydrated product (Figure 5c), the mineralized product exhibited more evident surface deposition, pore filling, and particle bridging (Figure 5f), consistent with its stronger carbonate-related XRD and FTIR responses and thermal characteristics. Carbonate precipitation in Mg-rich tailings and ultramafic wastes has previously been associated with surface deposition and cementation-related microstructural modification.

Overall, the SEM results indicate increased surface coverage, local pore filling, and interparticle contact after CO_2_ mineralization. When considered together with the carbonate-related XRD and FTIR responses [37,38,39], these morphological changes are consistent with the formation of carbonate-bearing products and associated microstructural reorganization. Changes in the Si–O–T region of the FTIR spectra, together with limited corresponding crystalline diffraction features, further suggest the possible presence of poorly crystalline or gel-like silicate/aluminosilicate products. However, the specific identity and contribution of these phases cannot be confirmed without direct compositional or structural characterization.

### 2.5. Pore-Structure Evolution and Mechanical Performance

The results presented in Section 2.1, Section 2.2, Section 2.3 and Section 2.4 demonstrate that CO_2_ mineralization induced composition-dependent changes in the carbonation behavior, thermal characteristics, crystalline phases, functional groups, and microstructure of the three synthetic tailing systems. The corresponding evolution of pore structure and mechanical performance is further discussed to clarify the structural and load-bearing responses to mineralization.

**Figure 6:**
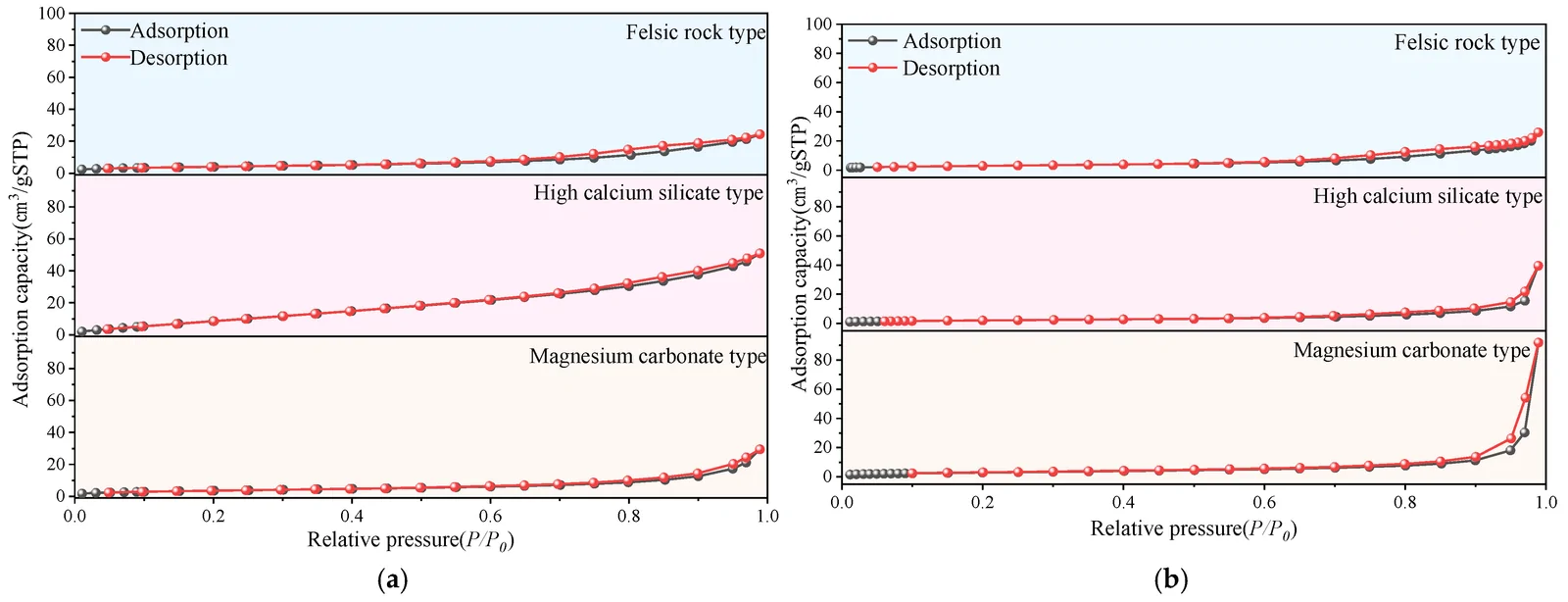
Nitrogen adsorption–desorption isotherms of the hydration and mineralization products from the three synthetic tailing systems: (**a**) hydration products and (**b**) mineralization products.

Nitrogen adsorption–desorption measurements were used to characterize the pore structures of the hydration and mineralization products. As shown in Figure 6, the adsorption–desorption isotherms differed among the three systems and changed after CO_2_ mineralization, indicating composition-dependent modification of the pore structure. The BET specific surface area decreased after mineralization for all three systems, from 14.48 to 10.78 m^2^/g for the felsic type, from 45.58 to 7.74 m^2^/g for the high-calcium silicate type, and from 13.09 to 10.63 m^2^/g for the magnesium—carbonate type. The decrease in specific surface area may be associated with surface coverage, particle aggregation, and the formation of mineralization products on particle surfaces.

**Figure 7:**
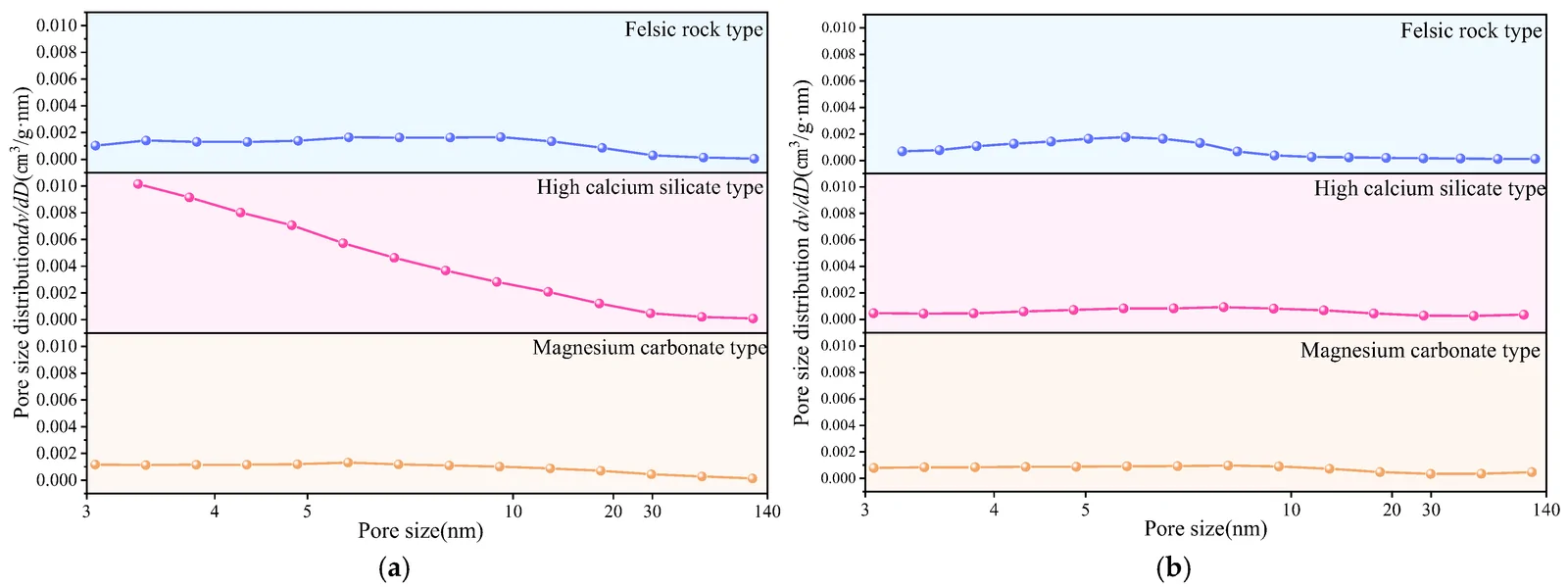
BJH pore-size distribution curves of the hydration and mineralization products from the three synthetic tailing systems: (**a**) hydration products and (**b**) mineralization products.

The BJH pore-size distributions [40,41] further showed that the pore structures were mainly characterized by mesopores and underwent different changes after mineralization (Figure 7). The hydrated products were primarily distributed within the 3–30 nm range. After mineralization, the felsic type showed a relatively narrow distribution mainly within approximately 4–10 nm, whereas the high-calcium silicate and magnesium-carbonate types exhibited an increased contribution from pores in the approximately 5–15 nm range. These changes indicate that CO_2_ mineralization modified the distribution of mesopores, with the specific response depending on the chemical composition of the synthetic tailing system.

**Figure 8:**
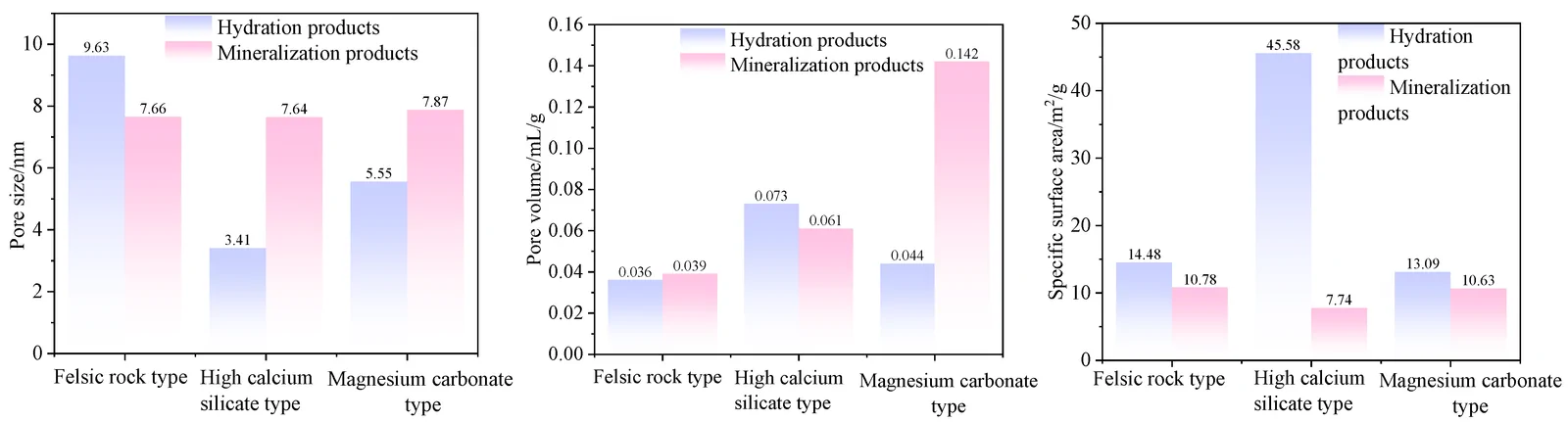
Average pore size, total pore volume, and specific surface area of the hydration and mineralization products from the three synthetic tailing systems.

The average pore size, total pore volume, and specific surface area are summarized in Figure 8. In the hydrated state, the average pore size followed the order of high-calcium silicate type < magnesium-carbonate type < felsic type, while the high-calcium silicate type exhibited the highest specific surface area and total pore volume, reaching 45.58 m^2^/g and 0.073 mL/g, respectively. After mineralization, the average pore sizes were within a relatively narrow range of approximately 7.5–7.9 nm. The total pore volume slightly increased from 0.036 to 0.039 mL/g for the felsic type, decreased from 0.073 to 0.061 mL/g for the high-calcium silicate type, and increased markedly from 0.044 to 0.142 mL/g for the magnesium-carbonate type. In contrast, the specific surface area decreased in all systems, with the high-calcium silicate type showing the largest reduction.

The above results indicate that CO_2_ mineralization did not lead to uniform pore densification. Instead, mineral precipitation, surface coverage, particle aggregation, and local pore rearrangement may have occurred simultaneously. In particular, the increased pore volume of the magnesium-carbonate type indicates that mineralization-induced structural rearrangement can occur even when abundant carbonate-bearing products are formed. Therefore, the pore-structure response should be interpreted as composition-dependent pore rearrangement rather than simple overall densification.

**Figure 9:**
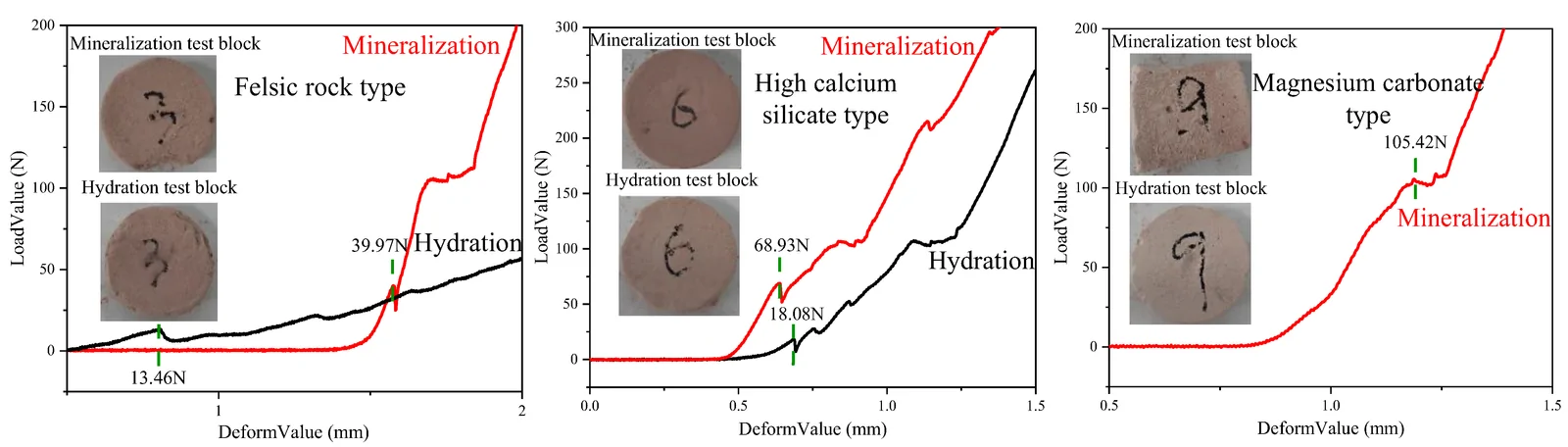
Representative photographs and compressive loading curves of hydrated and carbonated specimens from the three synthetic tailing systems.

The mechanical performance of the hydrated and carbonated specimens was subsequently evaluated. Representative photographs and compressive loading curves are presented in Figure 9. The hydrated specimens generally exhibited lower failure loads, whereas the carbonated specimens showed improved load-bearing behavior. The hydrated magnesium-carbonate specimens developed visible cracks before testing and therefore did not provide a valid mechanical baseline for comparison.

**Figure 10:**
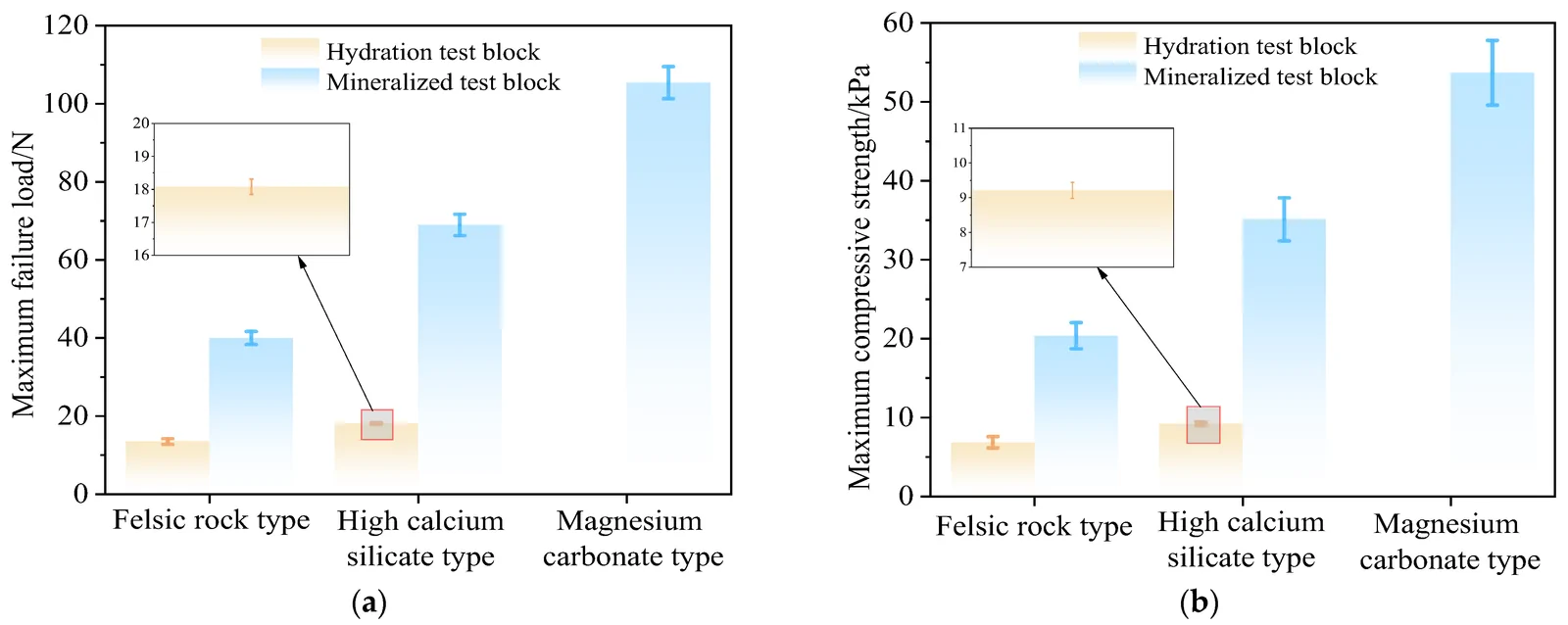
Failure load and apparent compressive strength of hydrated and carbonated specimens from the three synthetic tailing systems: (**a**) maximum failure load; (**b**) apparent compressive strength normalized by the cross-sectional area. Data are presented as mean ± standard deviation (*n* = 3); the hydrated magnesium-carbonate type was excluded from statistical comparison because no valid intact specimens were obtained.

The quantitative failure loads and apparent compressive strengths are summarized in Figure 10. After CO_2_ mineralization, the mean failure loads of the felsic, high-calcium silicate, and magnesium-carbonate types were 39.97 ± 1.67, 68.93 ± 2.74, and 105.42 ± 4.11 N, respectively. The corresponding apparent compressive strengths were 20.36 ± 0.85, 35.11 ± 1.40, and 53.69 ± 2.09 kPa, respectively. For the systems with valid hydrated mechanical baselines, the mean failure load increased from 13.46 ± 0.72 to 39.97 ± 1.67 N for the felsic type and from 18.08 ± 0.85 to 68.93 ± 2.74 N for the high-calcium silicate type, corresponding to enhancement factors of 2.97 and 3.81, respectively. The corresponding failure-load coefficients of variation ranged from 3.97% to 5.36%, indicating relatively low variability among the three replicate specimens. No enhancement factor was calculated for the magnesium-carbonate type because the hydrated specimens exhibited pre-existing cracking and therefore did not provide a valid mechanical baseline. Accordingly, the post-mineralization mechanical values for this system are presented as comparative indicators rather than as a paired strengthening ratio.

The improved mechanical response after carbonation is consistent with the formation of carbonate-bearing products and the increased surface coverage and interparticle contacts observed by SEM. However, because the total pore volume did not decrease uniformly among the three systems, the strength improvement cannot be attributed solely to overall densification. Instead, local precipitation-induced bonding, particle bridging, and pore-structure rearrangement likely contributed to the enhanced load-bearing capacity.

### 2.6. Mechanistic Interpretation of Mineralization-Induced Reinforcement

**Figure 11:**
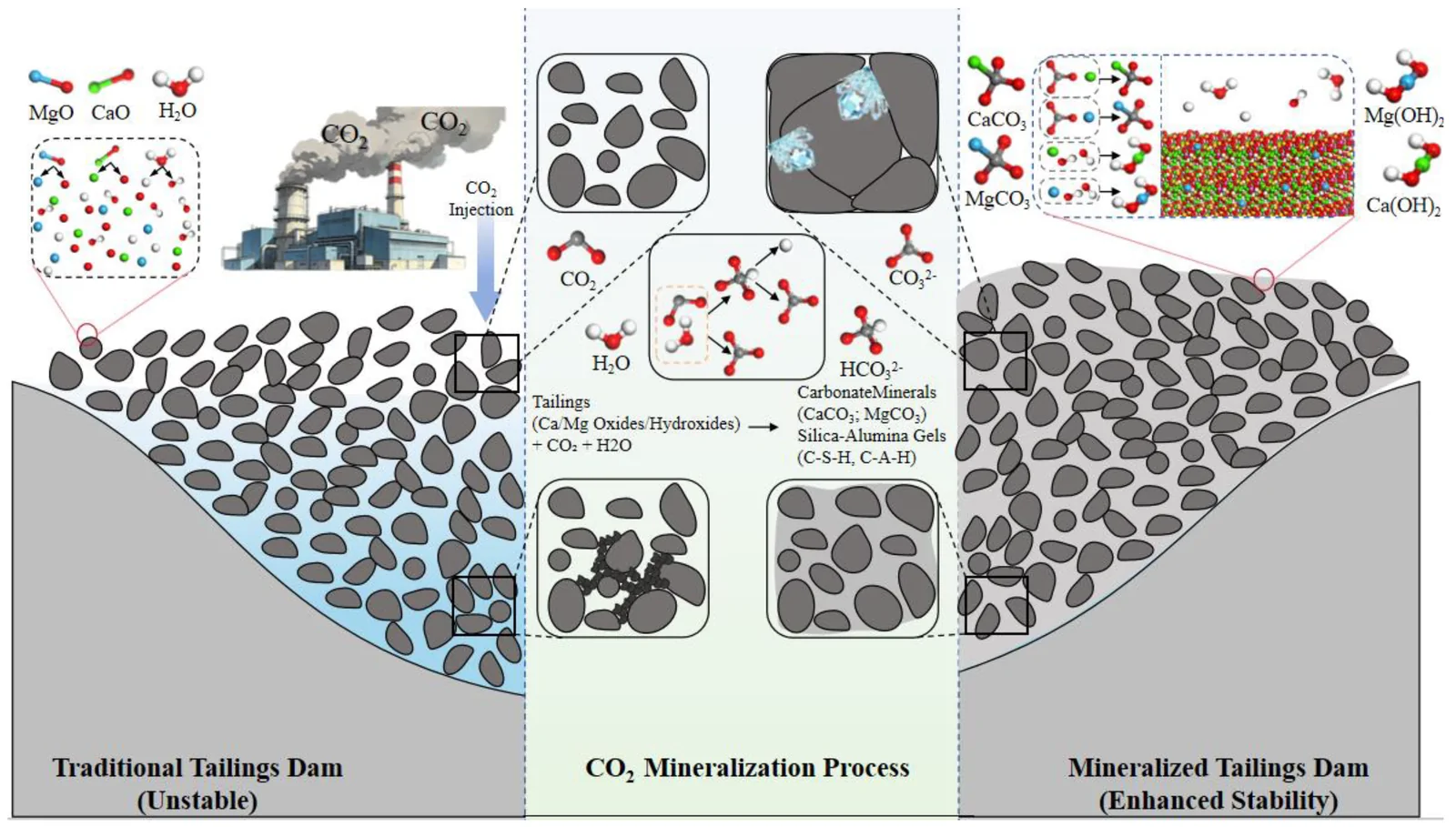
Proposed mechanism for mechanical performance improvement of tailings via CO_2_ mineralization.

The results indicate that mineral composition governs the mechanical response to CO_2_ mineralization by regulating alkalinity, the availability of reactive Ca and Mg species, and the subsequent precipitation and redistribution of reaction products. The proposed mechanism is illustrated in Figure 11.

During hydration and early CO_2_ exposure, the dissolution of reactive Ca- and Mg-bearing components generates alkaline conditions and releases metal ions into the aqueous phase. The availability and reactivity of these species differ among the three synthetic systems, resulting in distinct carbonation behaviors. Under alkaline conditions, dissolved CO_2_ is progressively converted to bicarbonate and carbonate species, which can react with available Ca^2+^ and Mg^2+^ to form carbonate-bearing precipitates. Thus, the mineral composition determines not only the extent of carbonate formation but also the potential locations and extent of precipitation within the particle network.

The resulting precipitates can modify interparticle contacts by depositing on particle surfaces, filling local voids, and forming bridges between adjacent particles. These changes may improve load transfer across particle contacts and thereby increase the resistance of the mineralized specimens to compressive loading. Possible poorly crystalline silicate/aluminosilicate products may provide an additional contribution where reactive Si- and Al-bearing components are available, although their specific phases and mechanical contribution cannot be confirmed from the present characterization.

The mechanical response further indicates that reinforcement does not require a uniform reduction in total pore volume. The high-calcium silicate type showed simultaneous pore-volume reduction and mechanical enhancement, whereas the magnesium-carbonate type achieved the highest post-mineralization strength despite an increase in total pore volume. This contrast suggests that the effectiveness of mineralization-induced reinforcement depends more strongly on the formation and distribution of load-transferring interparticle contacts than on overall pore-volume reduction alone.

Overall, the proposed mechanism can be summarized as mineral composition → alkalinity and ion availability → carbonation and precipitation → interparticle bonding and pore-network rearrangement → mechanical response. The relative importance of these processes varies with the chemical composition of the synthetic tailing system, providing a mechanistic explanation for the composition-dependent strengthening observed in this study [42,43].

### 2.7. Implications and Limitations of the Synthetic Model Systems

The comparative results demonstrate that the designed mineral composition strongly influenced the carbonation response and post-mineralization mechanical performance of the synthetic tailing systems. As summarized in Table 2, the felsic type exhibited the weakest carbonate-related response and the lowest post-carbonation mechanical performance, whereas the high-calcium silicate type showed pronounced pore-structure modification and the highest relative mechanical enhancement among the systems with valid paired mechanical data. The magnesium-carbonate type exhibited the longest alkaline plateau, the most pronounced carbonate-related XRD and FTIR responses, and the highest post-carbonation failure load and apparent compressive strength. These differences indicate that mineral composition affects not only the extent of carbonation but also the way in which mineralization-induced structural changes translate into mechanical reinforcement.

The comparison also highlights an important distinction between mineralization extent and mechanical reinforcement. The magnesium-carbonate type achieved the highest post-carbonation strength despite a substantial increase in total pore volume, whereas the high-calcium silicate type exhibited simultaneous reductions in specific surface area and total pore volume. This suggests that mechanical improvement cannot be attributed solely to overall pore-volume reduction and is more likely associated with the combined effects of carbonate-bearing precipitation, interparticle bonding, and pore-network reorganization, as discussed in Section 2.6.

The synthetic systems provide a controlled framework for isolating the influence of mineral composition, but their direct representation of natural tailings is limited. The materials were prepared from analytical-grade reagents and therefore do not fully reproduce the mineral assemblages, impurities, particle-size distributions, and surface characteristics of natural tailings. In addition, the use of highly soluble NaOH and KOH to represent Na- and K-bearing components may have increased the initial alkalinity and altered early-stage dissolution and carbonation behavior. The TG-DTG results should also be regarded as semi-quantitative indicators because an independent carbon balance was not established to determine absolute CO_2_ uptake. Furthermore, the possible poorly crystalline binding phases inferred from FTIR and SEM cannot be assigned to specific mineral phases without direct compositional or structural characterization.

The mechanical results should likewise be interpreted as comparative laboratory indicators rather than direct engineering parameters for natural tailings. Further validation using natural tailings, quantitative CO_2_ uptake measurements, direct phase and compositional characterization, and field-relevant mechanical and durability tests is required to assess the applicability of the proposed mineralization–reinforcement relationships.

## 3. Conclusions

The alkaline buffering behavior and carbonation response varied significantly among the three synthetic tailing systems. The duration of the pH plateau followed the order of magnesium-carbonate type > high-calcium silicate type > felsic type, whereas the initial pH-decline response followed the order of high-calcium silicate type > magnesium-carbonate type > felsic type. TG-DTG analysis showed that the magnesium-carbonate type exhibited the highest apparent carbonate-related mass-loss indicator, at approximately 40%. This value should be regarded as a semi-quantitative indicator of relative carbonate-bearing product formation rather than a direct measurement of absolute CO_2_ uptake.

XRD and FTIR results indicated the consumption or weakening of portlandite where present and the formation of carbonate-bearing phases during CO_2_ mineralization. The magnesium-carbonate type showed relatively stronger carbonate-related XRD and FTIR signals and the highest apparent TG mass-loss indicator, indicating a relatively greater abundance of carbonate-bearing products among the three synthetic systems. SEM observations revealed newly formed precipitate-like products on particle surfaces and within interparticle regions, accompanied by increased surface coverage and interparticle contact. BET-BJH analysis showed that the average pore sizes of the mineralized products converged to approximately 7–8 nm, mainly within the mesopore range, although the pore-structure evolution differed among the three systems.

Mechanical testing showed that the failure load and apparent compressive strength of the felsic and high-calcium silicate types increased after CO_2_ mineralization, with failure-load enhancement factors of 2.97 and 3.81, respectively. The mineralized magnesium-carbonate type exhibited the highest failure load and apparent compressive strength among the three mineralized systems, reaching 105.42 N and 53.69 kPa, respectively. However, no valid hydrated mechanical baseline was available for this system because of pre-existing cracks; therefore, no enhancement factor was calculated. Combined SEM, XRD, and FTIR evidence suggests that carbonate-bearing precipitates and possible amorphous or poorly crystalline silicate/aluminosilicate binding phases contributed to interparticle bonding and mechanical reinforcement.

Overall, the results provide mechanistic insights from synthetic model systems into composition-dependent CO_2_ mineralization behavior, pore-structure evolution, and mineralization-induced mechanical reinforcement. Further studies using natural tailings, complementary compositional analyses such as SEM-EDS, and quantitative carbon-balance measurements are required to determine the actual CO_2_ sequestration capacity and evaluate the engineering applicability, durability, and environmental stability of tailings-based mineralization systems.

## 4. Materials and Methods

### 4.1. Material Preparation

#### 4.1.1. Experimental Ratio

Synthetic tailings were prepared using a full-component reconstruction approach rather than being directly collected from natural tailings. Analytical-grade reagents with controlled particle size were used to isolate the effects of mineral chemical composition, particularly the CaO, MgO, and SiO_2_ contents, on CO_2_ mineralization and mechanical properties. This approach provides a simplified model system for establishing composition–structure–performance relationships while minimizing the influence of heterogeneous mineral morphology, particle-size distribution, and impurities commonly present in natural tailings.

The synthetic tailings were formulated according to the reported compositional ranges of different tailing types (Table 3) [44] The resulting full-component formulations are listed in Table 4. Because Na_2_O and K_2_O were not introduced directly as analytical-grade oxides, NaOH and KOH were used as their corresponding alkali sources. The substitution amounts were calculated according to the molar equivalence of Na and K between the corresponding oxides and hydroxides.

This substitution introduces Na and K in readily soluble forms and may therefore increase the initial alkalinity, accelerate the dissolution of reactive mineral components, and alter the relative contribution of hydration, dissolution, and carbonation pathways compared with natural tailings, in which Na and K are commonly incorporated within less-soluble mineral lattices. Consequently, the carbonation kinetics and reaction products observed in the present synthetic systems may differ from those of natural tailings. These effects should be considered when extrapolating the present results to field-derived materials.

The CaO, MgO, SiO_2_, Al_2_O_3_, Fe_2_O_3_, FeO, NaOH, and KOH used in this study were analytical-grade reagents with a particle size of 300 mesh. CO_2_ with a purity of 99.9% was supplied from compressed-gas cylinders, and deionized water was used throughout the experiments.

#### 4.1.2. Experimental Process

For the hydration experiments, the raw materials were weighed according to the formulations in Table 4 and transferred to a glass reactor. The pH of the reaction slurry was monitored continuously. After the pH stabilized, stirring was stopped and the hydration stage was terminated. The reacted slurry was then vacuum-filtered to obtain the wet solid products.

For mechanical testing, the filtered wet solids were placed into cylindrical molds and dried at 105 °C for 8 h to form self-supporting specimens without mechanical pressing. The detailed specimen preparation procedure for compressive testing is described in Section 4.5. The samples used for subsequent characterization were ground to 100 mesh and stored in sealed bags under vacuum.

**Figure 12:**
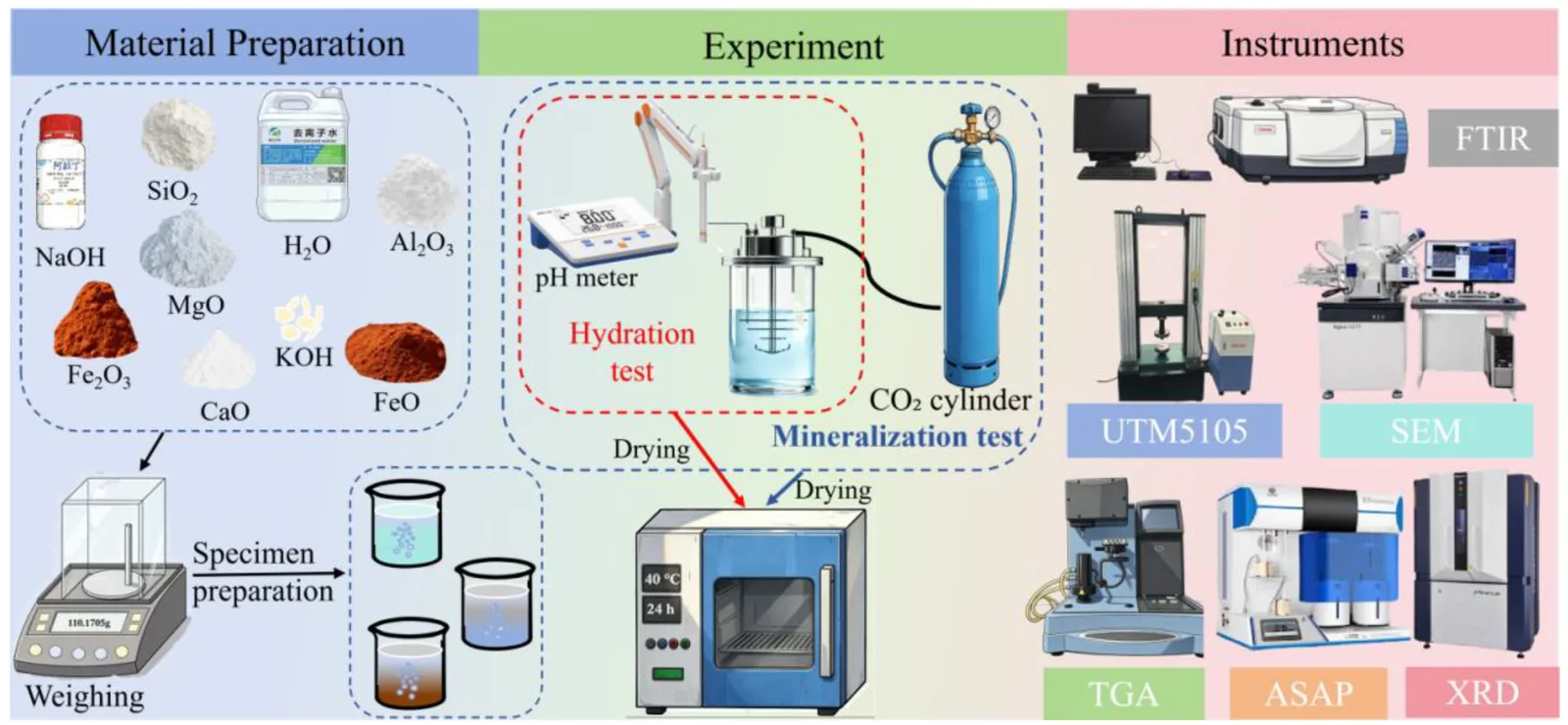
Schematic diagram of the CO_2_ mineralization experimental procedure.

The mineralization experimental procedure is shown in Figure 12. The raw materials were weighed according to Table 4 and uniformly mixed in the glass reactor using a glass rod. After the pH remained stable for 1 min, CO_2_ was introduced at a constant flow rate of 1 L/min, controlled using a gas valve and flowmeter. The pH variation during mineralization was continuously recorded. When the pH stabilized at approximately neutral conditions, the CO_2_ flow ceased, and themineralization stage was terminated.

The resulting slurry was filtered to obtain the wet solid products. For mechanical testing, the filtered wet solids were placed into cylindrical molds and dried at 105 °C for 8 h to form self-supporting specimens without mechanical pressing. The detailed specimen preparation procedure is described in Section 4.5. The samples used for subsequent characterization were ground to 100 mesh and stored in sealed bags under vacuum.

### 4.2. pH Meter and Thermogravimetric Test

The dynamic pH changes during the hydration and mineralization reactions were monitored using a pHS-3E pH meter (Shanghai INESA Scientific Instrument Co., Ltd., Shanghai, China). The thermal weight-loss characteristics of the raw materials and mineralized products were determined using a TA TGA 550 thermogravimetric analyzer (TA Instruments Inc., New Castle, DE, USA). The measurements were conducted under a nitrogen atmosphere from 20 to 850 °C at a heating rate of 10 °C/min.

### 4.3. FTIR and XRD Tests

The crystalline phases of the raw materials and mineralized products were analyzed using a Bruker D8 Advance X-ray diffractometer (Bruker AXS GmbH, Karlsruhe, Germany). The diffraction angle was scanned from 10° to 80° at a rate of 2°/min, with a slit width of 0.38 mm.

The chemical bonding characteristics of the raw materials and mineralized products were examined using a Thermo Scientific Nicolet iS5 Fourier-transform infrared spectrometer (Thermo Fisher Scientific Inc., Waltham, MA, USA). The samples were analyzed using the conventional KBr pellet method over a wavenumber range of 400–4000 cm^−1^.

### 4.4. SEM and Specific Surface Area and Pore Distribution Test

The morphology of the raw materials and mineralized products was examined using a Tescan MIRA LMS scanning electron microscope(TESCAN GROUP, a.s., Brno, Czech Republic). The SEM observations were used mainly to evaluate morphological changes before and after CO_2_ mineralization.

The specific surface area and pore-size distribution of the hydration and mineralization products were determined using a Micromeritics ASAP 2460 (Micromeritics Instrument Corporation, Norcross, GA, USA)automatic surface area and porosity analyzer based on nitrogen adsorption–desorption measurements. Before analysis, the samples were degassed at 120 °C.

### 4.5. Compressive Performance Test

After hydration and CO_2_ mineralization, the reacted slurries were filtered to obtain wet solid products. The filtered wet solids were transferred into cylindrical molds for specimen preparation. The specimens were formed by drying in molds rather than by mechanical pressing or compression molding; therefore, no controlled compaction pressure was applied during specimen preparation. Cylindrical specimens with a nominal diameter of 50 mm and a final height of 15 mm were used for compressive testing.

The wet solid products were uniformly filled into the cylindrical molds, and the upper surface was leveled before drying. The molded specimens were then dried at 105 °C for 8 h to obtain self-supporting solid specimens. After drying, the specimens were removed from the molds. Because slight differences in thickness or surface unevenness could occur after drying, the loading surfaces were carefully ground when necessary to obtain a consistent final height of approximately 15 mm and to ensure flat and parallel contact surfaces during compression testing. The actual diameter and height of each specimen were measured before testing.

For each hydration or mineralization condition, three replicate specimens were prepared and tested (*n* = 3). The apparent compressive strength results are reported as the mean ± standard deviation (SD). Only intact specimens without visible pre-existing cracks were used for the statistical calculation.

The compressive tests were conducted using a UTM5105 microcomputer-controlled electronic universal testing machine (Sansi Zongheng, Shenzhen, China). Each specimen was centrally positioned between the loading platens and loaded under displacement control at a constant rate of 1 mm/min until failure. The maximum load was recorded as the failure load. The apparent compressive strength was calculated according to Equations (7) and (8):

(7) $$R=\frac{F}{S}$$

(8) $$S=\frac{\pi D^{2}}{4}$$

where *R* is the compressive strength (MPa), *F* is the maximum failure load (N), *S* is the loading area (mm^2^), and *D* is the specimen diameter (mm). For a nominal diameter of 50 mm, the calculated loading area was 1963.5 mm^2^.

## Figures and Tables

**Table 1:** Main FTIR band assignments used for interpreting hydration and CO_2_ mineralization products.

Wavenumber/cm^−1^	Assignment	Possible Interpretation
3400–3650	O–H stretching	Hydroxide-bearing products, such as portlandite-like phases
1630–1650	H–O–H bending	Adsorbed or bound water
1400–1500	v_3_ CO_3_^2−^ vibration	Carbonate-bearing products
870–880	v_2_ CO_3_^2−^ vibration	Out-of-plane bending of carbonate groups
710–730	v_4_ CO_3_^2−^ vibration	Carbonate-bearing phases
950–1100	Si–O–T stretching, T = Si or Al	Silicate/aluminosilicate frameworks or possible poorly crystalline silicate/aluminosilicate binding phases
460–520	Si–O bending	Silicate/aluminosilicate structural vibrations

**Table 2:** Summary of mineralization, pore-structure, and mechanical indicators of the three synthetic tailing systems.

Indicators	Felsic Type	High Calcium Silicate Type	Magnesium Carbonate Type
pH plateau duration/s	~300	~650	~2000
Apparent TG mass-loss indicator/%	~36	~37	~40
Carbonate-related XRD response	Weakest	Pronounced	Most pronounced
Carbonate-related FTIR response	Weakest	Pronounced	Most pronounced
Possible poorly crystalline binding phases	Limited indication	Moderate indication	Relatively pronounced indication
BET specific surface area, hydration → mineralization/m^2^/g	14.48 → 10.78	45.58 → 7.74	13.09 → 10.63
Total pore volume, hydration → mineralization/mL/g	0.036 → 0.039	0.073 → 0.061	0.044 → 0.142
Carbonated failure load/N	39.97	68.93	105.42
Carbonated apparent compressive strength/kPa	20.36	35.11	53.69
Load enhancement factor	2.97	3.81	—

Note: The TG-DTG mass-loss values are semi-quantitative indicators of apparent mass loss associated with carbonate-bearing products and do not represent absolute CO_2_ uptake. The load enhancement factor of the magnesium-carbonate type was not calculated because a valid hydrated mechanical baseline was not obtained.

**Table 3:** Chemical composition ranges of the three representative tailing types.

Tailings Type	Main Chemical wt%							
	SiO_2_	Al_2_O_3_	Fe_2_O_3_	FeO	MgO	CaO	Na_2_O	K_2_O
Felsic rock type	65~80	12~18	0.5~2.5	1.5~2.5	0.5~1.5	0.5~4.5	3.5~5	2.5~5.5
High calcium silicate type	35~55	5~12	3~5	2~15	5~8.5	20~30	0.5~1.5	0.5~2.5
Magnesium carbonate type	1~5	0.5~2	0.1~3	0~0.5	17~24	26~35	trace	trace

**Table 4:** Typical metal tailings full-component compounding system.

Tailings Type	Ingredient Content/g								
	SiO_2_	Al_2_O_3_	Fe_2_O_3_	FeO	MgO	CaO	NaOH	KOH	H_2_O
Felsic rock type	34.93	7.23	0.72	0.96	0.48	1.20	4.10	1.93	515.54
High calcium silicate type	22.57	4.26	2.01	4.26	3.39	12.54	0.50	0.75	502.88
Magnesium carbonate type	2.63	1.10	1.36	0.22	17.97	26.73	0.00	0.00	500.00

## Data Availability

The data supporting the findings of this study are available from the corresponding author upon reasonable request.
